# Arteriovenous extracorporeal lung assist allows for maximization of oscillatory frequencies: a large-animal model of respiratory distress

**DOI:** 10.1186/1471-2253-8-7

**Published:** 2008-11-14

**Authors:** Ralf M Muellenbach, Julian Kuestermann, Markus Kredel, Amélie Johannes, Ulrike Wolfsteiner, Frank Schuster, Christian Wunder, Peter Kranke, Norbert Roewer, Jörg Brederlau

**Affiliations:** 1 University of Wuerzburg, Department of Anaesthesiology; University hospital Wuerzburg; Oberduerrbacherstr. 6; 97080 Wuerzburg, Germany

## Abstract

**Background:**

Although the minimization of the applied tidal volume (VT) during high-frequency oscillatory ventilation (HFOV) reduces the risk of alveolar shear stress, it can also result in insufficient CO_2_-elimination with severe respiratory acidosis. We hypothesized that in a model of acute respiratory distress (ARDS) the application of high oscillatory frequencies requires the combination of HFOV with arteriovenous extracorporeal lung assist (av-ECLA) in order to maintain or reestablish normocapnia.

**Methods:**

After induction of ARDS in eight female pigs (56.5 ± 4.4 kg), a recruitment manoeuvre was performed and intratracheal mean airway pressure (mPaw) was adjusted 3 cmH_2_O above the lower inflection point (Plow) of the pressure-volume curve. All animals were ventilated with oscillatory frequencies ranging from 3–15 Hz. The pressure amplitude was fixed at 60 cmH_2_O. At each frequency gas exchange and hemodynamic measurements were obtained with a clamped and de-clamped av-ECLA. Whenever the av-ECLA was de-clamped, the oxygen sweep gas flow through the membrane lung was adjusted aiming at normocapnia.

**Results:**

Lung recruitment and adjustment of the mPaw above Plow resulted in a significant improvement of oxygenation (p < 0.05). Compared to lung injury, oxygenation remained significantly improved with rising frequencies (p < 0.05). Normocapnia during HFOV was only maintained with the addition of av-ECLA during frequencies of 9 Hz and above.

**Conclusion:**

In this animal model of ARDS, maximization of oscillatory frequencies with subsequent minimization of VT leads to hypercapnia that can only be reversed by adding av-ECLA. When combined with a recruitment strategy, these high frequencies do not impair oxygenation

## Background

The current strategy for conventional mechanical ventilation (CMV) in patients with acute respiratory distress syndrome (ARDS) is to prevent further lung injury which is a result of alveolar over-distension and the repetitive collapse and reopening of damaged alveoli [1-3]. Lung-protective ventilation using low tidal volume (VT) has been shown to reduce ventilator-induced lung injury (VILI) and mortality in patients with ARDS [2,4,5]. Theoretically high-frequency oscillatory ventilation (HFOV) achieves all goals of a lung-protective ventilatory mode. It provides the application of extremely small VTs combined with a relatively high mean airway pressure (mPaw). Thereby a more uniform lung recruitment can be achieved while the risk of tidal overdistension is minimized [6,7]. In adults with ARDS, HFOV can be safely applied and may improve gas exchange compared with CMV [8]. During HFOV, VT and thus carbon dioxide (CO_2_)-elimination are directly related to the applied pressure amplitude (ΔP) and the inspiratory/expiratory ratio and are inversely related to the oscillatory frequency- in other words: The lower the oscillatory frequency, the higher the resulting VT [9].

In adult HFOV trials the applied frequencies varied from 3–6 Hz and, in an attempt to maximize CO_2_-elimination, a ΔP from 60–90 cmH_2_O was used [10,11]. In these settings the resulting VTs resemble those during conventional lung-protective ventilation, thereby antagonizing the potential advantages of HFOV [9,12]. Although it was shown that higher oscillatory frequencies (15 Hz) might result in less neutrophil infiltration compared with lower frequencies (5 Hz) in a small animal model of respiratory distress, in adults the application of higher frequencies may lead to insufficient CO_2_-elimination with severe respiratory acidosis [13-15].

Recently, a new pumpless arteriovenous extracorporeal lung assist system (av-ECLA) has been introduced into clinical practice. It consists of a low-resistance membrane lung (iLA, NovaLung^®^, Hechingen, Germany) interposed into a simple arteriovenous shunt between the femoral artery and vein [16]. Depending on the diameter of the cannulas, the cardiac output (CO) and the mean arterial pressure (MAP), the transmembraneous blood flow equals 20–30% of the CO [17]. With these flow rates near total extracorporeal CO_2_-removal could be achieved in animal models of respiratory failure, thereby allowing a more lung protective conventional ventilation strategy [18,19].

While significant reductions in minute ventilation and VTs can be obtained during the combination of av-ECLA and conventional mechanical ventilation, optimal oscillatory frequencies remain to be determined. The current study was performed to evaluate the effects of different oscillatory frequencies on CO_2_-elimination with and without av-ECLA in a large-animal model of ARDS. We hypothesized that the application of high oscillatory frequencies, and thereby minimization of VT and alveolar stretch, requires the combination of HFOV with av-ECLA in order to maintain or re-establish normocapnia.

## Methods

### Animal Preparation

The study was conducted in accordance with the National Institutes of Health guidelines for ethical animal research and was approved by the Laboratory Animal Care and Use Committee of the District of Unterfranken, Germany. The experiments were performed on eight healthy female pigs (56.5 ± 4.4 kg). Anaesthesia and instrumentation were carried out as previously described [20].

Shortly after intramuscular premedication with ketamin (10 mg/kg,) an intravenous line was obtained and the animals were anaesthesized with continuous infusion of 5–10 mg/kg thiopental and 0.01 mg/kg/h fentanyl throughout the experiment. Neuromuscular block was achieved by continuous infusion of 0.1 mg/kg/h pancuronium.

The trachea was intubated with a cuffed 8.5 mm ID endotracheal tube with an additional side lumen ending at the tip (Rueschelit^®^, Ruesch, Kernen, Germany). The lung was mechanically ventilated in the pressure controlled ventilation mode (EvitaXL, Dräger Medical, Lübeck, Germany) starting with a PEEP of 5 cmH_2_O, a VT of 6 ml/kg, a respiratory rate (RR) of 30/min and an inspiratory to expiratory ratio (I:E) of 1:1. The inspiratory oxygen fraction (FiO_2_) remained 1.0 throughout the entire experiment. The core temperature was maintained at 38.0 ± 0.5°C during the experiment by using a heating pad.

The animals were instrumented with a central venous line (Arrow International, Reading, PA, USA), an arterial catheter (Vygon, Ecouen, France) and a pulmonary artery catheter (831F75, Edwards Lifescience, Irvine, CA, USA). The position of the pulmonary artery catheter was verified by typical waveforms. After systemic heparinization (400 U/kg, Liquemin, Roche, Reinach, Switzerland) a 17 -French cannula was inserted into the right femoral artery and a 17-French cannula (NovaLung^®^, Hechingen, Germany) into the left femoral vein using Seldinger technique and ultrasound guidance (SonoSite 180 Plus, SonoSite Inc., Botell, WA). A low-resistance pumpless extracorporeal lung assist device (iLA, NovaLung^®^, Hechingen, Germany) was primed with heparinized isotonic saline (150 ml) and connected to the femoral cannulas. The av-ECLA circuit was afterwards occluded with tubing clamps. The activated clotting time (ACT II, Medtronic, Minneapolis, MN) was measured hourly and maintained between 300–400 seconds with continuous heparin infusion (1000 – 2000 U/h).

### Experimental Protocol

**Figure 1:**
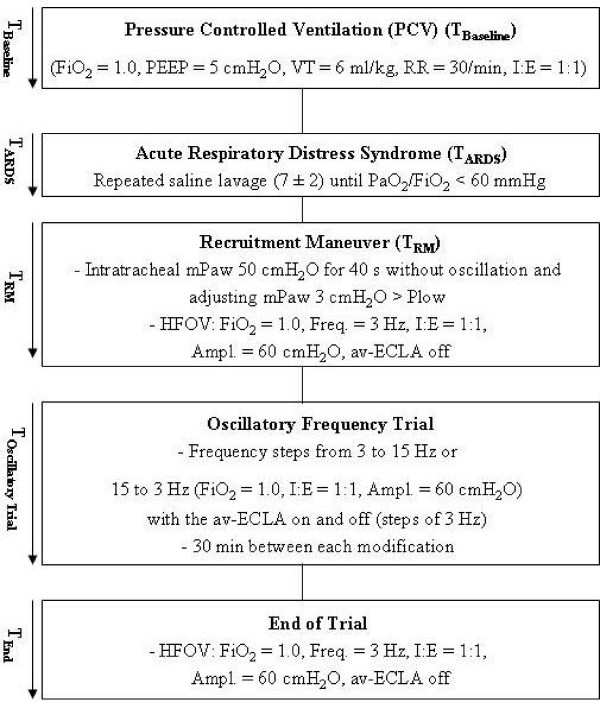
**Study protocol and time course.*** PCV*, Pressure controlled ventilation; *HFOV*, high-frequency oscillatory ventilation; *FiO*_2_, fraction of inspired oxygen; *PEEP*, positive endexpiratory pressure; *VT*, tidal volume; *RR*, respiratory rate; *I:E*, inspiratory:expiratory-ratio; *mPaw*, mean pulmonary airway pressure; *Plow*, lower inflection point of the pressure-volume curve; *PaO*_2_, arterial oxygen partial pressure; *PaCO*_2_, carbon dioxide partial pressure; av-*ECLA*, arteriovenous extracorporeal lung assist device.

The study protocol of the experiment is outlined in Fig. 1. After instrumentation, the animals were stabilized for 30 min and baseline measurements (T_Baseline_)were obtained. Severe ARDS (T_ARDS_) was induced by bilateral pulmonary lavages with 30 ml/kg isotonic saline (38°C), repeated every 10 minutes until PaO_2 _decreased to less than 60 mmHg and remained stable for 60 minutes with unchanged ventilator settings. An average of 7 ± 2 lavages with approximately 12.000 ml saline per animal was necessary for ARDS induction. During induction all animals were ventilated with PCV (FiO_2 _= 1.0, PEEP = 5 cmH_2_O, VT = 6 ml/kg, RR = 30/min, I:E = 1:1). After the induction of ARDS the lower inflection point (Plow) of the thoracic pressure-volume curve (PV) was determined in each animal. Accordingly, the animals were switched from PCV to HFOV (Sensor Medics 3100 B, Yorba Linda, CA, USA) and a lung recruitment manoeuvre (RM) was performed. This was realized by increasing the mean airway pressure (mPaw) to 50 cmH_2_O without oscillations for 40 s. The intratracheal mPaw was then adjusted 3 cmH_2_O above Plow. Afterwards, HFOV was started with an oscillatory frequency of 3 Hz and RM measurements were obtained (T_RM_). Subsequently, the animals were ventilated with oscillatory frequencies ranging from 3 Hz to 15 Hz. The frequency was altered in steps of 3 Hz in an incremental or decremental order. The starting oscillatory frequency was randomly assigned (3 Hz or 15 Hz to each half of the animals) to rule out a time effect in gas exchange and hemodynamic measurements due to recovery of the injured lung. At each oscillatory frequency, measurements were obtained with the av-ECLA circuit opened and closed. A 30-minute period was given for equilibration between each modification prior to data collection. The total study period lasted approximately 6 hours. As the av-ECLA circuit was de-clamped, normocapnia was attempted by adjusting the sweep gas flow through the membrane lung (0 – 10 l oxygen/min), which was applied via a calibrated O_2_-flowmeter (Draeger, Luebeck, Germany). Ventilator settings during HFOV were set as follows throughout the trial: FiO_2 _= 1.0, bias flow = 30 l/min, oscillatory pressure amplitude = 60 cmH_2_O, I:E = 1:1. At the end of the study the oscillatory frequency was set at 3 Hz with the av-ECLA circuit clamped. A continuous colloid infusion (Voluven^® ^6% HES 130/0.4, Fresenius Kabi, Bad Homburg, Germany) was administered in addition to a balanced electrolyte solution to maintain a mean arterial pressure (MAP) > 70 mmHg, which is mandatory to maintain sufficient blood flow through the membrane lung. Before killing the animals with an overdose of thiopental and embutramid mebezonium iodide (T 61^®^, Intervet, Unterschleissheim, Germany) the PV curve was repeatedly measured.

### Measurements

All measurements were performed during HFOV at different oscillatory frequencies (3, 6, 9, 12 or 15 Hz) with the av-ECLA circuit opened or closed (Fig. 1). For hemodynamic monitoring pressure transducers referenced to atmospheric pressure at the mid-thoracic level (Combitrans^®^, Braun, Melsungen, Germany) and a modular monitor system (Servomed^®^, Hellige, Freiburg i. Br., Germany) were used. MAP, mean pulmonary artery pressure (MPAP), central venous pressure (CVP) and pulmonary artery occlusion pressure (PCWP) were transduced. The heart rate (HR) was traced by the electrocardiogram. Threefold injections of 10-ml aliquots of ice cold saline into the right atrium were used for pulmonary artery catheter-based cardiac output measurements (Explorer^®^, Edwards Lifescience, Irvine, CA, USA).

Blood flow through the extracorporeal circuit was measured continuously with an ultrasonic flow probe (HT 110^®^, Transonic, Ithaca, NY, USA). Arterial, post av-ECLA and mixed venous blood samples were immediately analyzed for PO_2_, PCO_2 _and pH using standard blood gas electrodes (ABL 505^®^, Radiometer, Bronshoj, Denmark). In each sample, hemoglobin and oxygen saturation were measured using spectrophotometry (OSM3^®^, Radiometer, Bronshoj, Denmark). Arterial and mixed venous oxygen contents (ml/dl) and the pulmonary shunt fraction (Qs/Qt) were calculated using standard formulas.

Intratracheal mean airway pressure (mPaw) was measured at the tubes tip using an air filled pressure transducer referenced to atmospheric pressure (PM8050^®^, Draeger, Luebeck, Germany). Quasistatic pressure-volume curves of the respiratory system were obtained using the in- and expiratory low-flow pressure-volume manoeuvre of the Evita XL^® ^ventilator. The start pressure was set at 0 cmH_2_O and the peak inspiratory pressure at 50 cmH_2_O with a flow rate of 4 l/min and a maximum volume of 2 l.

### Statistical analysis

In each animal, both the starting oscillatory frequency (3 Hz or 15 Hz in each half of the animals; variation of frequency in steps of 3 Hz every 30 min) and the order of opening and closing the av-ECLA at each frequency were randomized. The data of the incremental and decremental oscillatory trial were pooled and analyzed using SigmaStat 2.03 (Systat Software Inc., Point Richmond, USA). The data were tested for normal distribution using the Kolmogorov-Smirnov test. Values are reported as mean ± SD. One and two-way analysis of variance (ANOVA) for repeated measurements were used for data analysis. Student-Newman-Keuls post hoc test was used for comparison of significant ANOVA results. Adjustment for multiple testing was performed. P values < 0.05 were considered significant.

## Results

**Figure 2:**
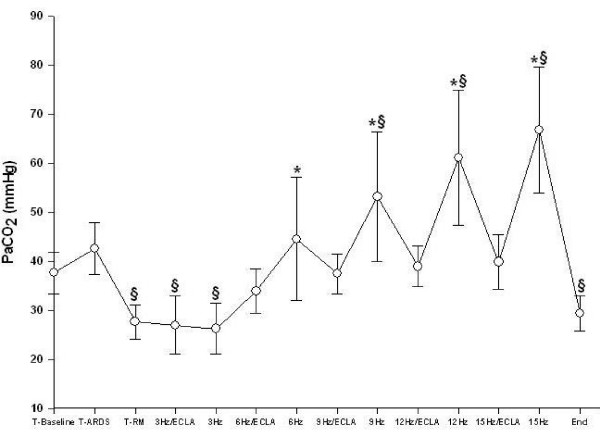
**Arterial carbon dioxide partial pressure (PaCO_2_) during different oscillatory frequencies with the arteriovenous extracorporeal lung assist device (ECLA) opened and closed.** After recruitment and switching ventilation to HFOV the PaCO_2 _was significantly lower compared with lung injury (T_ARDS_) (§p < 0.05). The PaCO_2 _were significantly reduced from 6 to 15 Hz with the ECLA opened (* p < 0.05).

**Figure 3:**
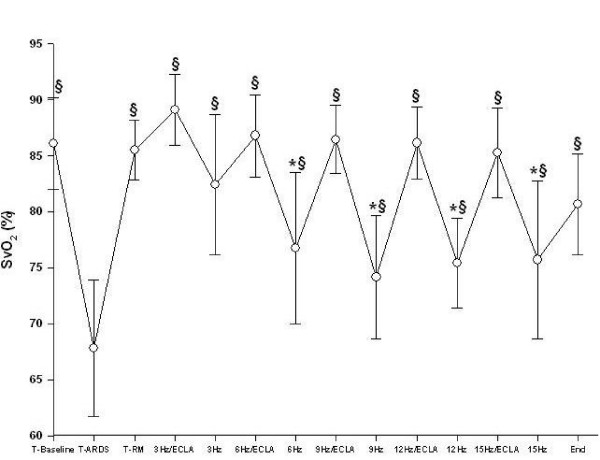
**Mixed venous oxygen saturation (SvO_2_) during different oscillatory frequencies with the arteriovenous extracorporeal lung assist device (ECLA) opened and closed.** After recruitment and switching ventilation to HFOV the SvO_2 _was significantly improved compared with lung injury (T_ARDS_) (§p < 0.05). The SvO_2 _were significantly increased from 6 to15 Hz with the ECLA device opened (* p < 0.05).

**Figure 4:**
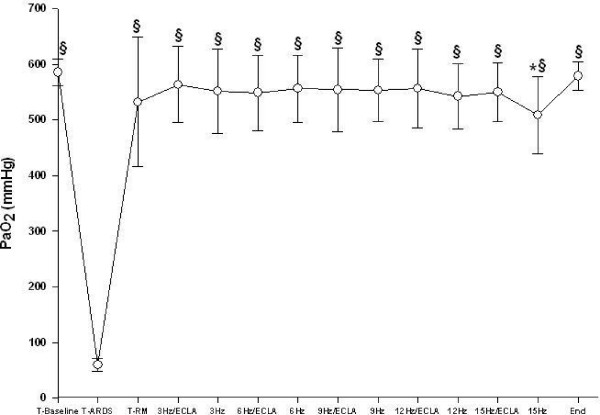
**Arterial oxygen partial pressure (PaO_2_) during different oscillatory frequencies with the arteriovenous extracorporeal lung assist device (ECLA) opened and closed.** After lung recruitment and switching ventilation to HFOV the PaO_2 _was significantly higher compared with lung injury (T_ARDS_) (§p < 0.05). PaO_2 _remained significantly improved with rising oscillatory frequencies compared with T_ARDS _(§p < 0.05). The PaO_2 _was significantly increased at 15 Hz with the ECLA opened (* p < 0.05).

All animals survived the complete study period. Gas exchange and hemodynamic parameters did not differ between the animals randomized for the incremental or decremental oscillatory trial before and after lung injury. After saline lavage, all animals developed severe respiratory failure (Table 1, Fig. 2, 3, and 4). Detailed data regarding gas exchange, respiratory parameters, hemodynamics and av-ECLA related parameters, are presented in Tables 1 and 2.

**Table 1:** Gas exchange, respiratory and arteriovenous extracorporeal lung assist parameters, before and after lung injury, post recruitment and during the time course

	**T**_**Baseline**_	**T**_**ARDS**_	**T**_**RM**_	**3 Hz/ECLA**	**3 Hz**	**6 Hz/ECLA**	**6 Hz**
RR	30+/-0	30+/-0	180+/-0§	180+/-0§	180+/-0§	360+/-0§	360+/-0§
mPaw	9.3+/-0.4§	13.2+/-1.4	22.6+/-1.1§	22.6+/-1.1§	22.6+/-1.1§	22.6+/-1.1§	22.6+/-1.1§
FiO_2_	1.0+/-0	1.0+/-0	1.0+/-0	1.0+/-0	1.0+/-0	1.0+/-0	1.0+/-0
pHa	7.45+/-0.09	7.41+/-0.06	7.55+/-0.04§	7.6+/-0.04§	7.58+/-0.07§	7.48+/-0.07	7.4+/-0.08*
PaO_2_	585+/-24§	60+/-12	532+/-116§	563+/-69§	551+/-75§	549+/-68§	556+/-60§
PaCO_2_	37.7+/-4.2	42.7+/-5.3	27.7+/-3.5§	27.1+/-6§	26.3+/-5.3§	34+/-4.6	44.6+/-12.5*
SvO_2_	86.1+/-4.1§	67.9+/-6.1	85.5+/-2.7§	89.1+/-3.2§	82.4+/-6.3§	86.8+/-3.7§	76.8+/-6.7§*
DO_2_	906+/-295	739+/-251	919+/-223	920+/-198	706+/-189*	804+/-192	593+/-211*
VO_2_	197+/-35	169+/-30	214+/-34§	192+/-31	184+/-31	182+/-39	182+/-38
Qs/Qt	0.07+/-0.02§	0.54+/-0.09	0.12+/-0.09§	0.11+/-0.07§	0.1+/-0.06§	0.11+/-0.06§	0.07+/-0.04§*
Plow	5.8+/-0.5§	19.6+/-1.1					
P_ECLA_O_2_				564+/-36		574+/-45	
SGF				0+/-0		2.4+/-2.3	
EBF				1.4+/-0.2		1.4+/-0.2	
EBF/CO				21+/-6		25+/-6	

	**9 Hz/ECLA**	**9 Hz**	**12 Hz/ECLA**	**12 Hz**	**15 Hz/ECLA**	**15 Hz**	**End**

RR	540+/-0§	540+/-0§	720+/-0§	720+/-0§	900+/-0§	900+/-0§	180+/-0§
mPaw	22.6+/-1.1§	22.6+/-1.1§	22.6+/-1.1§	22.6+/-1.1§	22.6+/-1.1§	22.6+/-1.1§	22.6+/-1.1§
FiO_2_	1.0+/-0	1.0+/-0	1.0+/-0	1.0+/-0	1.0+/-0	1.0+/-0	1.0+/-0
pHa	7.46+/-0.05	7.34+/-0.08*	7.45+/-0.05	7.29+/-0.08*	7.43+/-0.06	7.26+/-0.07*	7.54+/-0.05§
PaO_2_	554+/-74§	553+/-56§	557+/-71§	542+/-59§	550+/-53§	508+/-70§S*	579+/-25§
PaCO_2_	37.5+/-4	53.2+/-13.3§*	39+/-4.2	61.1+/-13.7§*	39.9+/-5.5	66.8+/-12.7§*	29.5+/-3.6§
SvO_2_	86.5+/-3.1§	74.2+/-5.5§*	86.1+/-3.2§	75.5+/-4§*	85.3+/-4§	75.7+/-7§*	80.7+/-4.5§
DO_2_	764+/-152	538+/-118*	767+/-110	558+/-135*	819+/-194	590+/-169*	616+/-76
VO_2_	177+/-38	181+/-32	180+/-34	181+/-36	194+/-33	182+/-29	178+/-38
Qs/Qt	0.1+/-0.07§	0.06+/-0.03§*	0.1+/-0.07§	0.06+/-0.04§*	0.1+/-0.05§	0.08+/-0.04§	0.08+/-0.03§
Plow							20.7+/-3.5
P_ECLA_O_2_	608+/-36		628+/-22		620+/-28		
SGF	3.9+/-4.1		5+/-4		5+/-4.1		
EBF	1.5+/-0.2		1.4+/-0.2		1.4+/-0.3		
EBF/CO	27+/-6		26+/-2		24+/-4		

Data expressed as means ± standard deviation; *ECLA*, arteriovenous extracorporeal lung assist; *ARDS*, acute respiratory distress syndrome; *PRM*, post recruitment; *RR*, respiratory rate per minute; *mPaw*, mean pulmonary airway pressure in cmH_2_O; *FiO*_*2*_, fraction of inspired oxygen; *PaO*_*2*_, arterial oxygen partial pressure in mmHg; *PaCO*_*2*_, arterial carbon dioxide partial pressure in mmHg; *SvO*_*2*_, mixed venous oxygen saturation; *DO*_*2*_, oxygen; delivery in l/min/m^2^; *VO*_*2*_, oxygen consumption in l/min/m^2^;*Qs/Qt*, pulmonary shunt fraction; *Plow*, lower inflection point in cmH_2_O;*P*_*ECLA*_*O*_*2*_, partial oxygen pressure behind lung assist in mmHg; SGF, Sweep Gas Flow in l/min; EBF, ECLA Blood Flow in l/min; *CO*, cardiac output; * *p *< 0.05 ECLA vs. No ECLA; §*p *< 0.05 vs. T_ARDS_.

**Table 2:** Haemodynamic variables before and after lung injury, post recruitment and during the time course

	**T**_**Baseline**_	**T**_**ARDS**_	**T**_**RM**_	**3 Hz/ECLA**	**3 Hz**	**6 Hz/ECLA**	**6 Hz**
HR/min	72+/-18	75+/-20	78+/-24	84+/-19	75+/-18*	75+/-19	71+/-17
MAP	66+/-3§	81+/-8	79+/-8	74+/-9	83+/-13*	78+/-9	86+/-14*
CVP	4.3+/-3.2	4.8+/-2	11.5+/-1.9§	10.3+/-1.5§	10.4+/-1.3§	9.9+/-1.6§	10.8+/-1.8§
MPAP	13+/-3,3	15.6+/-2.1	22.3+/-2.3§	23.5+/-3.5§	22.1+/-3.4§	24.1+/-3§	25+/-3.9§
PCWP	6.1+/-2	7.3+/-2	14.6+/-3.2§	13.1+/-1.6§	12.3+/-1.3§	13.1+/-2§	12.8+/-2.1§
CO	5.6+/-1.5	6.8+/-2	6.7+/-1.4#	6.8+/-2	5.3+/-1.8*	6+/-1.9	4.4+/-2§*
SVR	926+/-264	950+/-232	845+/-228#	801+/-253	1220+/-449*	968+/-260	1506+/-511§*
PVR	98+/-20	106+/-29	94+/-46#	134+/-61	168+/-75	159+/-66	269+/-168§*

	**9 Hz/ECLA**	**9 Hz**	**12 Hz/ECLA**	**12 Hz**	**15 Hz/ECLA**	**15 Hz**	**End**

HR/min	69+/-17	60+/-15*	71+/-16	62+/-14*	69+/-19	63+/-21	74+/-13
MAP	80+/-13	84+/-14	77+/-11	80+/-13	77+/-10	75+/-15	87+/-10
CVP	11+/-1.7§	11+/-2.3§	10.9+/-1.6§	10.6+/-1.2§	11.3+/-1.6§	10.1+/-1.8§	10.4+/-1.4§
MPAP	25.1+/-1.8§	25.3+/-3§	23.8+/-1.9§	25.1+/-3.5§	23.3+/-3§	25+/-3,9§	23.5+/-3.6§
PCWP	13.9+/-1.5§	12.3+/-1.3§*	13.3+/-1.3§	12.8+/-1.8§	14.6+/-2.1§	13.3+/-1.3§*	12.6+/-0.9§
CO	5.7+/-1.5	4+/-1§*	5.6+/-0.7	4.1+/-0.9§*	6+/-1.1	4.3+/-1.1§*	4.5+/-0.6§
SVR	1008+/-226	1542+/-405§*	949+/-140	1385+/-285§*	900+/-142	1224+/-262*	1404+/-330§
PVR	171+/-54	282+/-100§*	154+/-44	242+/-58§*	119+/-38	218+/-63§*	202+/-88§

Data expressed as means ± standard deviation; *ECLA*, arteriovenous extracorporeal lung assist; *ARDS*, acute respiratory distress syndrome; *PRM*, post recruitment; *HR*, heart rate; *MAP*, mean arterial blood pressure in mmHg; *MPAP*, mean pulmonary artery pressure in mmHg; *PCWP*, pulmonary artery occlusion pressure in mmHg; *CO*, cardiac output in l/min; *SVR*, systemic vascular resi stance in dyne·s/cm^5^; *PVR*, pulmonary vascular resi stance in dyne·s/cm^5^; * *p *< 0.05 ECLA vs. No ECLA; §*p *< 0.05 vs. T_ARDS_; # *p *< 0.05 T_RM _vs. End.

### Pulmonary Gas Exchange

Lung recruitment and adjusting the mPaw 3 cmH_2_O above Plow during HFOV led to a significant improvement of PaO_2_, SvO_2_, and Qs/Qt-ratio (Table 1, Fig. 3, and 4, p < 0.05). At T_RM_, at 3 Hz/ECLA and at 3 Hz, PaCO_2 _was significantly reduced and pH was significantly increased compared with T_ARDS _(Table 1, p < 0.05). PaO_2 _and Qs/Qt-ratio remained significantly improved with rising oscillatory frequencies compared with T_ARDS _(Table 1 and Fig. 4, p < 0.05). Pulmonary shunt fraction was significantly higher between 6 Hz and 12 Hz during HFOV compared with HFOV/av-ECLA (Table 1, p < 0.05). PaCO_2 _was significantly reduced and pH was significantly higher between 6 Hz and 15 Hz with the av-ECLA opened as compared with HFOV alone (Table 1 and Fig. 2, p < 0.05). SvO_2 _was significantly higher during HFOV/av-ECLA compared with HFOV with all oscillatory frequencies applied (Fig. 3, p < 0.05). At the end of the study, gas exchange parameters were not significantly different from T_RM _(Table 1).

### Hemodynamics and Oxygen Delivery

After lung recruitment, CVP, MPAP, PCWP and VO_2 _were significantly elevated vs. T_ARDS _(Table 1 and 2, p < 0.05). CVP, MPAP and PCWP remained elevated compared with T_ARDS _throughout the study; significant differences between HFOV and HFOV/av-ECLA could be found in the PCWP at 9 Hz and 15 Hz (Table 2, p < 0.05). Heart rate and mean arterial pressure did not change significantly compared with T_ARDS _at different oscillatory frequencies. At 3 Hz and 9 Hz, the HR was significantly higher during HFOV/av-ECLA (Table 2, p < 0.05). MAP was significantly elevated during HFOV at 3 Hz and 6 Hz. SVR was significantly decreased and CO and DO_2 _were significantly increased during HFOV/av-ECLA compared with HFOV from 3 Hz to 15 Hz (Table 1 and 2, p < 0.05). PVR was significantly higher during HFOV compared with HFOV/av-ECLA between 6 Hz and 15 Hz (Table 2, p < 0.05). VO_2 _did not differ between study groups (Table 1).

### Arteriovenous extracorporeal lung assist device

Blood gas analysis directly behind the membrane lung resulted in an oxygen partial pressure from 564 ± 36 mmHg to 628 ± 22 mmHg. After applying higher oscillatory frequencies, normocapnia was achieved by increasing the O_2_-sweep gas flow from 2.4 ± 2.3 l/min to 5.0 ± 4.1 l/min. Av-ECLA-blood flow was constant with 1.4 ± 0.2 l/min throughout the study, resulting in an av-ECLA-blood flow/CO-ratio of 21 ± 6% to 27 ± 6%.

## Discussion

The major findings of this study are twofold: (1) With oscillatory frequencies from 9–15 Hz normocapnia could only be maintained if HFOV was combined with av-ECLA. (2) After lung recruitment and setting the mPaw 3 cmH_2_O above Plow, a sustained improvement in oxygenation was detectable even when oscillatory frequencies up to 15 Hz were used.

Conventional mechanical ventilation during severe ARDS can aggravate the progress of lung damage, especially when high tidal volumes are applied and repeated tidal collapse and reopening of damaged lung units occur. Limitation of the VT to 6 ml/kg of the predicted body weight, while keeping the lungs open with sufficient positive end-expiratory pressure (PEEP) can significantly reduce mortality in ARDS [3,4]. Further minimization of the VT combined with the application of high mean airway pressures may be achieved using HFOV [6]. Thereby, inspiratory overdistension and expiratory de-recruitment can be avoided [21]. Recently, it has been shown in a large-animal model of ARDS, that HFOV reduces histological signs of lung inflammation and messenger RNA expression of interleukin-1-beta in lung tissue compared to conventional lung-protective ventilation [22]. In most clinical studies using HFOV in adults, oscillatory frequencies between 3–6 Hz and pressure amplitudes between 60–90 cmH_2_O have been used [10,11]. If hypercapnia was evident during HFOV then oscillatory frequencies were decreased to 3 Hz and amplitudes were increased up to 90–100 cmH_2_O. However, oscillatory frequencies below 4 Hz combined with maximum pressure amplitudes resulted in tidal volumes comparable to conventional lung-protective ventilation, thereby antagonizing the potential advantages of HFOV [12].

The idea of decoupling ventilation and oxygenation was introduced by Gattinoni et al [23-25]: Extracorporeal CO_2_-elimination was provided by a veno-venous perfusion route with 20–30% of the cardiac output, whereas oxygenation was maintained by mechanical ventilation. Several animal and human trials showed the feasibility of reducing ventilator requirements during pump-driven extracorporeal CO_2_-removal [23,24,26,27]. However, despite technical advances of these systems, several side-effects and complications impede its benefits, e.g., pump-induced traumatisation of blood cells, plasma leakage of oxygenator membranes and activation of the coagulation and inflammatory system.

Pumpless extracorporeal lung assist became feasible with the introduction of low-resistance membrane lungs that could be interposed into a simple arteriovenous shunt between the femoral artery and vein [28]. With the patient's heart as the driving force, the transmembranous blood flow is up to 25–30% of the CO, thereby allowing comparable CO_2_-elimination and reductions in ventilatory support achieved during pumpdriven extracorporeal CO_2_-removal [19]. In an adult sheep model of respiratory failure, av-ECLA allowed significant reductions in VTs (from 15 ± 1.6 to 3 ± 1.5 ml/kg), peak inspiratory pressures (40 ± 2.1 to 20 ± 7.5 cmH_2_O) and minute ventilation (10 ± 1.4 to 0.5 ± 0 L/min) [19].

The goal of this large-animal study of ARDS was to evaluate the effects of HFOV during different oscillatory frequencies on CO_2_-elimination with and without the application of av-ECLA. Our results clearly demonstrate that normocapnia was not achieved and respiratory acidosis developed if higher oscillatory frequencies (> 9 Hz) were used without av-ECLA. Although 'permissive hypercapnia' might be one element of a lung-protective ventilation strategy because of its potential beneficial effects, such as suppression of lung inflammation, the safe level of hypercapnia and the respiratory acidosis remains unknown [29]. In addition, there are clinical situations where hypercapnia is contraindicated, e.g. in patients with decreased cerebral compliance [30].

It has now been accepted, that VT-reduction is the key factor to improving outcome and to protecting the lungs from further damage, whereas the optimal VT remains unclear [4,31]. In our study, the implementation of av-ECLA allowed the use of maximal oscillatory frequencies (up to 15 Hz), similar to those applied during neonatal HFOV, without the risk of hypercapnia or respiratory acidosis. Although not measured in this study, with the settings chosen, the resulting VTs can safely be assumed to be below 1 ml/kg of the predicted body weight [16,32]. Nevertheless, mere VT-reduction during conventional ventilation leads to low peak pressure ventilation favouring further lung de-recruitment [33]. Very recently, Dembinski and co-workers compared the use of av-ECLA and VT-reduction to 3 ml/kg with a conventional low-tidal volume (6 ml/kg) ventilation strategy in a large-animal model of ARDS [34]. After 24 hours, the VT-reduction in the av-ECLA group resulted in a significant impairment of oxygenation and an increase of the pulmonary shunt fraction compared with the CMV group. However, the PEEP levels used in this study were very low and insufficient in preventing lung de-recruitment. In ARDS patients ventilated with low tidal volumes, de-recruitment is usually reversed by a recruitment manoeuvre or prevented by an adequate PEEP level [33]. Adjusting the mPaw level for optimal lung recruitment remains difficult during HFOV. Usually mPaw during HFOV is set 3 to 5 cmH_2_O higher than the mPaw used during CMV [21]. In an animal model of respiratory distress, it was shown that setting the mPaw 6 cmH_2_O above the lower inflection point of the pressure-volume curve yielded the best oxygenation [21]. Using a similar recruitment strategy, we found sustained improvements of oxygenation, even with maximum oscillatory frequencies. Since VT can be minimized when HFOV is combined with av-ECLA, an optimal recruitment of lung volume appears to be essential in this setting.

The study design implies the following limitations: First, we used an ARDS model based on surfactant-depletion. In adults, not surfactant deficiency, but alveolar flooding is the predominant mechanism in ARDS development with surfactant-depleted lungs responding better to lung recruitment [35]. Second, the Sensormedics 3100 B oscillator is a pressure cycling machine that can neither control nor directly measure VTs. Hence, in this study no direct statement regarding the applied VTs can be made. However, in a recent observational study VT was measured directly during HFOV in ARDS patients: It was shown, that the tidal volumes during HFOV with frequencies between 5 and 12 Hz are between 0.8 and 3.3 ml/kg of the predicted body weight [12]. Third, the study protocol did not allow alterations of the pressure amplitude and I:E-ratio in order to improve CO_2_-elimination. Both parameter settings refer to previous experimental large-animal studies of HFOV and clinical practice [11,22,36]. However, if maximal oscillatory frequencies without av-ECLA were used, only moderate hypercapnia and respiratory acidosis developed. Therefore, one might argue, that without av-ECLA CO_2_-elimination could have been improved by adjusting the pressure amplitude and I:E-ratio to 90–100 cmH_2_O and 1:2, respectively. On the other hand, with near total CO_2_-removal due to extracorporeal lung assist, the applied pressures and accordingly the applied VT could have been further reduced, thereby minimizing VT and alveolar shear stress. Finally, av-ECLA allows for decoupling of ventilation and oxygenation. Therefore, the application of more lung-protective ventilator settings is not only possible, but should also be examined. However, it was not the purpose of this study to examine the effect of higher frequency HFOV on lung damage.

Despite the potential benefits with regard to lung protective ventilation strategies, different complications are described during av-ECLA. These included infections, ischemia of the lower limb after cannulation, bleeding and cannula thrombosis [16]. In addition, the device should not be used in patients with cardiogenic shock, heparin-induced thrombocytopenia and severe peripheral arterial occlusive disease.

## Conclusion

In conclusion, we demonstrate in a large-animal model of ARDS, that the combination of HFOV and av-ECLA guarantees normocapnia even with maximal oscillatory frequencies of 9 to 15 Hz. In this context, av-ECLA can be interpreted as a tool to unmask the entire lung protective potential of HFOV. Minimization of the applied VT does not only limit tidal overdistension and volutrauma, but also facilitates lung recruitment and prevents the lungs from atelactrauma since the application of higher mean airway pressures is possible. Thereby, oxygenation can be maintained. Histologic and immunologic data are needed in order to prove the lung protective effects of the proposed therapeutic concept.

In a further trial, it should be evaluated whether the combination of HFOV with maximal oscillatory frequencies and the av-ECLA result in further lung-protection when compared to conventional lung-protective ventilation.

## Abbreviations

ARDS: Acute respiratory distress syndrome; av-ECLA: Arteriovenous extracorporeal lung assist; CMV: Conventional mechanical ventilation; CO: Cardiac output; CO_2_: carbon dioxide; CVP: Central venous pressure; DO_2_: Oxygen delivery; FiO_2_: Inspiratory oxygen fraction; HFOV: High-frequency oscillatory ventilation; HR: Heart rate; I:E: Inspiratory to expiratory ratio; MAP: Mean arterial pressure; mPaw: Mean airway pressure; MPAP: Mean pulmonary artery pressure; PaCO_2_: Arterial carbon dioxide tension; PaO_2_: Arterial oxygen tension; PCWP: Pulmonary artery wedge pressure; PEEP: Positive end-expiratory pressure; PIP: Peak inspiratory pressure; Plow: Lower inflection point; PRM: Post recruitment measurements; PV: Pressure-volume curve; PVR: Pulmonary vascular resistence; Qs/Qt: pulmonary shunt fraction; RM: Recruitment manoeuvre; RR: Respiratory rate; SvO_2_: Mixed venous oxygen tension; SVR: System vascular resistance; VILI: Ventilator-induced lung injury; VO_2_: Oxygen consumption; VT: Tidal volume.

## Competing interests

The authors declare that they have no competing interests.

## Authors' contributions

RMM designed the study, collected data, and drafted the manuscript. JK collected the data and helped to write the manuscript. MK and AJ performed the statistical analysis. UW collected data. FS participated in the design of the study. CW, PK and NR helped to interpret the results and write the manuscript. JB drafted the manuscript and participated in the design of the study. All authors read and approved the final manuscript.

## Pre-publication history

The pre-publication history for this paper can be accessed here:


